# Determination of Optimal Heart Rate Variability Features Based on SVM-Recursive Feature Elimination for Cumulative Stress Monitoring Using ECG Sensor

**DOI:** 10.3390/s18072387

**Published:** 2018-07-23

**Authors:** Dajeong Park, Miran Lee, Sunghee E. Park, Joon-Kyung Seong, Inchan Youn

**Affiliations:** 1 Biomedical Research Institute, Korea Institute of Science and Technology (KIST), 5, Hwarang-ro 14-gil, Seongbuk-gu, Seoul 02792, Korea; dajeong@kist.re.kr (D.P.); leemiran7461@gmail.com (M.L.); 2 Department of Bioengineering, University of Pennsylvania, Philadelphia, PA 19104, USA; park.estelles@gmail.com; 3 Department of Bio-convergence Engineering, Korea University, 145, Anam-ro, Seongbuk-gu, Seoul 02841, Korea; jkseong@korea.ac.kr; 4 Department of KHU-KIST Converging Science and Technology, Kyung Hee University, 26, Kyungheedae-ro, Dongdaemun-gu, Seoul 02447, Korea

**Keywords:** heart rate variability, cumulative stress, electrocardiogram, stress monitoring, support vector machine-recursive feature elimination

## Abstract

Routine stress monitoring in daily life can predict potentially serious health impacts. Effective stress monitoring in medical and healthcare fields is dependent upon accurate determination of stress-related features. In this study, we determined the optimal stress-related features for effective monitoring of cumulative stress. We first investigated the effects of short- and long-term stress on various heart rate variability (HRV) features using a rodent model. Subsequently, we determined an optimal HRV feature set using support vector machine-recursive feature elimination (SVM-RFE). Experimental results indicate that the HRV time domain features generally decrease under long-term stress, and the HRV frequency domain features have substantially significant differences under short-term stress. Further, an SVM classifier with a radial basis function kernel proved most accurate (93.11%) when using an optimal HRV feature set comprising the mean of R-R intervals (*mRR*), the standard deviation of R-R intervals (*SDRR*), and the coefficient of variance of R-R intervals (*CVRR*) as time domain features, and the normalized low frequency (*nLF*) and the normalized high frequency (*nHF*) as frequency domain features. Our findings indicate that the optimal HRV features identified in this study can effectively and efficiently detect stress. This knowledge facilitates development of in-facility and mobile healthcare system designs to support stress monitoring in daily life.

## 1. Introduction

Increased awareness of the negative effects of stress on personal health has concomitantly increased interest in routine stress monitoring. Stress can be caused by various factors stemming from physical (e.g., allergies, fatigue, or poor sleep), psychological (e.g., conflicts, trauma, or work demands), or environmental (e.g., noise, crowds, or disasters) influences [1]. When the body is temporarily exposed to stress, the body responds by activating the sympathetic nervous system with simultaneous withdrawal of the parasympathetic nervous system [2]. However, chronically accumulated stress can lead to dysfunction of the stress response system. Autonomic dysfunction has been closely associated with serious health problems such as hypertension, cardiovascular disease, and depression [3,4]. In general, stress symptoms appear only after serious health problems have occurred. Therefore, routine stress monitoring is essential for managing stress in a timely manner and providing proper feedback to avoid serious health issues.

The electrocardiogram (ECG) biosignal has been widely used for stress monitoring. ECG analysis provides a feasible option for routine monitoring because it can be supported in real-time mobile or wearable device applications. Many previous studies have used heart rate variability (HRV) from the ECG biosignal for monitoring stress, and a variety of HRV features have been used to evaluate stress as it relates to autonomic nervous system (ANS) activity [5]. However, several experimental studies have demonstrated that not all HRV features provide accurate stress-related information. For example, decreased high frequency power (*HF*), increased low frequency power (*LF*), and *LF/HF* have been identified as potential indicators of stress [6]. However, Kim et al. [7] suggested that the HRV frequency power could be influenced by personal traits such as the individual’s preference in coping with stress. Similarly, Lee et al. [8] identified heart rate (*HR*) as the strongest indicator of driving stress and found that other time domain features tended to change based on personal traits. Furthermore, since the involvement of irrelevant features in the monitoring system might increase the computational time and lead to poor performance [9], determination of the optimal features is essential for the monitoring system. Therefore, identifying the optimal HRV features related to cumulative stress is important for routine stress monitoring.

The goal of this study was to determine the optimal set of HRV features that would accurately detect cumulative stress and subsequently improve the efficiency of routine stress monitoring. We first investigated the effects of cumulative stress on various HRV features via an experimental study. We conducted experiments using a rodent model, which is widely used as a stress and depression model, since the use of a human model has rigid restrictions and ethical rules for chronic physiological stress exposure. Next, we determined an optimal HRV feature set using support vector machine-recursive feature elimination (SVM-RFE). Specifically, we calculated a ranking criterion for HRV features in both time and frequency domains using SVM-RFE and determined the optimal feature set based on the results of six classifiers.

The remainder of this paper is organized as follows. Section 2 discusses related work. Section 3 describes experimental approaches employed in determining the optimal HRV features for cumulative stress monitoring. Section 4 presents the results obtained regarding the effects of short- and long-term stress on various HRV features based on experimental methods and the optimal HRV feature set based on SVM-RFE methods. Section 5 discusses the implications of these results in the context of the broader state of knowledge. Section 6 presents concluding remarks.

## 2. Related Work

Various researchers have attempted to determine the optimal or important HRV features for stress monitoring. Endukuru et al. [10] used analysis of variance (ANOVA) and the unpaired *t*-test to demonstrate that some HRV features—the mean of heart rates (*mHR*), the standard deviation of R-R intervals (*SDRR*), the square root of the mean squared difference between adjacent R-R intervals (*RMSSD*), the number of N-N intervals that differ by more than 50 ms (*NN50*), the proportion of N-N intervals that differ by more than 50 ms (*pNN50*), *LF*, *HF*, and *LF/HF*—were sensitive in response to a mental stress test. Vargas-Luna et al. [11] used statistical analysis to confirm that two spectral features of HRV—*LF* and *HF*—significantly differed between rest and mental tasks. Similarly, Borchini et al. [12] used the analysis of covariance (ANCOVA) linear regression models to find a significant association of *LF* and *HF* with prolonged work stress.

In several studies, a feature selection approach such as the filter [13,14] and wrapper method [13,15,16] was used for determining optimal HRV features. Aigrain et al. [15] evaluated the predictive power of various multimodal features by investigating the composition of the best feature subset and showed that the HR values (maximum and variation) and the amplitude of HR (maximum, mean, and variation) provided the best prediction among features related to ECGs. Ollander et al. [13] used both the filter and wrapper method to search multimodal features related to stress for detecting driving stress.

In the determination of optimal HRV features, most previous studies considered acute stress in response to a specific stressor (mental or driving load) rather than cumulative stress. Although a few studies of HRV features have been performed on accumulated stress, these studies analyzed the effects of HRV features on stress rather than identifying optimal features for a monitoring system. In our study, we analyzed the effects of cumulative stress and determined the optimal features for cumulative stress monitoring using SVM-RFE as the wrapper method for feature selection.

## 3. Materials and Methods

### 3.1. Experimental Study

#### 3.1.1. Animals

Forty-five male rats (Sprague-Dawley rat, 250 g, Charles River Laboratories International, Incheon, Korea) were used in this study. We randomly divided the rats into three groups (n = 15 per group): Control, short-term stress (SS), and long-term stress (LS) groups. The rats had free access to food and water and were kept at controlled temperature (22 ± 2 °C) with a 12 h light-dark cycle. All experiments were performed in accordance with the recommendations for the care and use of laboratory animals by the Ethical Committee of the Korea Institute of Science and Technology (2016-013).

#### 3.1.2. Cumulative Stress Protocol

**Figure 1:**
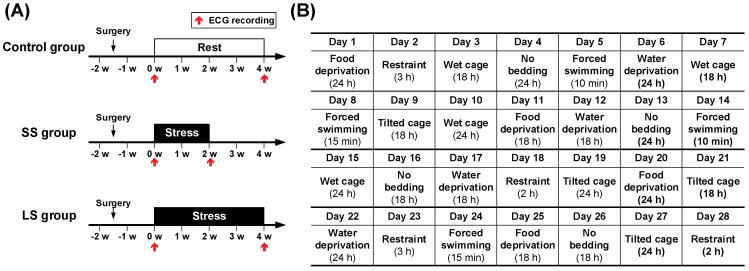
Experimental design used in this study. Based on the timeline of the experimental protocols, after 10 days of recovery from surgery, rats in the short-term stress (SS) or long-term stress (LS) groups were exposed to two or four weeks of stress, respectively, while rats in the control group rested. (**A**) At the baseline and the end of the two- and four-week stress periods, electrocardiograms (ECGs) were recorded for each group. (**B**) Based on the schedule of stress procedures, rats in the two stress groups (SS and LS) were exposed to unpredictable mild stress every day using seven randomly selected stressors.

We used the chronic mild stress (CMS) procedure to create the environments of cumulative stress in animal model [17]. Based on reversal results after three weeks of the CMS treatments in previous study [18], we exposed the SS and LS groups to unpredictable mild stress for either short-term (two weeks) or long-term (four weeks) periods, respectively (Figure 1A). The stress procedure is based on the protocol used in previous studies [19,20]. The rats in each of the stress groups (SS or LS groups) were exposed, once daily, to one of the following randomly selected stressors: Water deprivation (24 or 18 h), food deprivation (24 or 18 h), 45° tilted cage (18 h), wet cage (18 h), no bedding (18 h), restraint (2 or 3 h), and forced swimming (10 or 15 min). These stressors were randomly exposed to all rats of stress groups for each stress procedure (Figure 1B). The rats in the control group took their rest in each house for experimental periods.

### 3.2. Radio-Telemetry System

**Figure 2:**
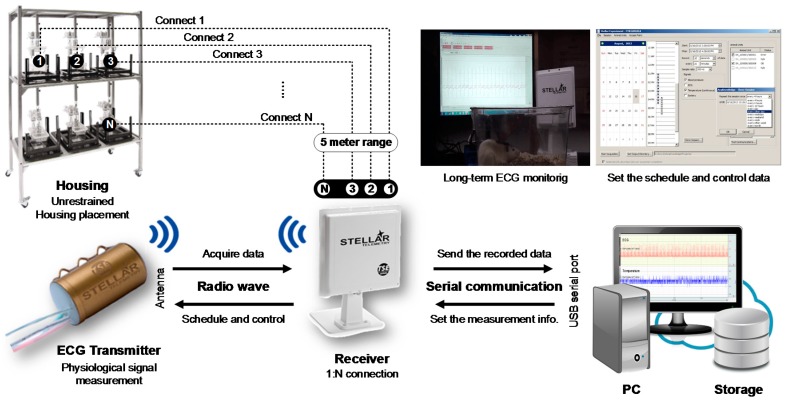
Schematics of radio-telemetry system for the ECG recording. The ECGs, which were measured from the transmitter ECG sensor, were wirelessly transferred to the receiver through radio communication. The data stored in the receiver can be monitored and processed on the PC via the USB serial communication.

In order to obtain electrocardiograms (ECGs) in unrestrained rats for long-term periods, we used ECG telemetry system (TSE system, Chesterfield, MO, USA), consisting of a receiver and implantable ECG transmitter sensors (Figure 2). Since the receiver could acquire the data wirelessly from a number of animals in longer distance (up to 5 m), we could perform the data acquisition simultaneously from all rats in each cage without restraint. The implantable ECG sensor was suitable for implantation into rat’s abdominal cavities due to its small volume (6 cm^3^) and light weight (11 g). Furthermore, since the battery life was up to 12 months in case of animals with weight more than 170 g, this system was ideal for our experimental protocol over more than 5 weeks. The sampling rate of ECG recording could be flexibly selected up to 1 kHz. In our study, ECGs were recorded at sampling rate of 1 kHz for 5 min, referring the previous study that time and frequency domain HRV analysis required at least a sampling rate of l kHz for rats [21]. The ECG data was transferred via USB serial communication to the PC configured using AcqKnowledge 4.4 (BIOPAC Systems, Inc., Goleta, CA, USA).

### 3.3. Transmitter Implant Surgery

**Figure 3:**
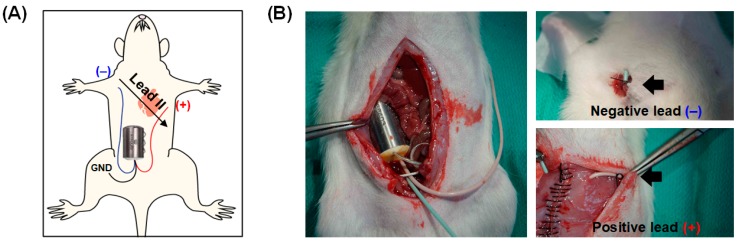
Rat model with surgically implanted transmitter to record electrocardiograms. (**A**) Implantable transmitter placement in a lead II configuration and (**B**) transmitter implant surgical process in which the transmitter body was positioned in the rat’s abdominal cavity with the positive and negative electrodes fixed to the left caudal rib and right pectoral muscle, respectively.

Transmitters with negative and positive electrodes were surgically implanted in all of the rats. Figure 3 depicts this surgical implant process. Briefly, while each rat was under anesthesia, the telemetry transmitter was implanted in the rat’s abdominal cavity. The electrodes were placed in the modified lead II configuration. The positive and negative electrodes were fixed to the left caudal rib and right pectoral muscle, respectively. To avoid irritation of the tissue, the two leads were ensured to lie flat against the muscle. The reference lead was attached in the lower right quadrant on the inside of the abdominal muscle. Following surgery, the rats were allowed 10 days to recover before stress experiments were initiated.

### 3.4. HRV Analysis

ECGs were recorded at the baseline (pre-test) and at the end of the experimental period (post-test). The recorded ECGs were divided into two trials, each comprising a 150-s window without overlap. An R-R tachogram was generated by detecting the R peak in each ECG trial. The HRV features were subsequently calculated in the time and frequency domains from the R-R tachogram. The HRV time domain features included the mean of R-R intervals (*mRR*), *mHR*, *SDRR*, the coefficient of variance of R-R interval (*CVRR*), *RMSSD*, and the proportion of consecutive N-N intervals that differ by more than 5 ms (*pNN5*). The HRV frequency domain features were calculated from the R-R tachograms using fast Fourier transformation (FFT; Welch’s periodogram with a Hamming window of 512 points and 50% overlap). The frequency bands used in this study were the low frequency (*LF*; 0.1 to 1.0 Hz) and high frequency (*HF*; 1.0 to 3.5 Hz) bands. The HRV frequency domain features included the absolute values of each band power and the ratio between the power bands. To obtain normal distributions, *HF*, *LF*, and the ratio of *LF* and *HF* were converted into natural logarithms (*ln LF*, *ln HF*, and *ln (LF/HF)*). In addition, the *LF* and *HF* were presented in normalized units (*nLF* and *nHF*).

### 3.5. Support Vector Machine-Recursive Feature Elimination

**Figure 4:**
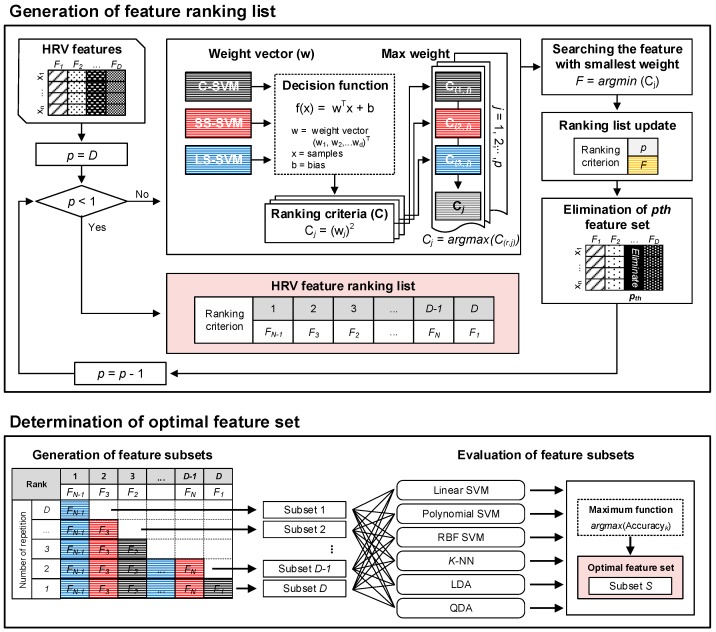
Schematic outline of the support vector machine-recursive feature elimination (SVM-RFE) process, which includes generating a ranking criterion for heart rate variability (HRV) features (**top**) and using it to determine the optimal feature set based on the results of six classifiers (**bottom**).

We used the SVM-RFE method for determining the optimal features. The SVM-RFE method performs feature selection using sequential backward elimination based on SVM [22]. In the construction of the SVM model, the weights of the features were calculated. This method has been proven to be more scalable and more efficient than other feature selection methods [9]. Figure 4 shows a schematic outline of the SVM-RFE process, in which a ranking criterion for HRV features was generated and used to determine the optimal feature set based on the results of six classifiers. We used the ECG signal of the dataset acquired at end of the experimental procedure in each group for determining the optimal features. Before initiating this process, we normalized all data involving the HRV features over a 0 to 1 range using the min-max method.

#### 3.5.1. Ranking Criterion Generation

We first developed individual multiclass support vector machines (SVMs) to identify HRV features for the control, SS, and LS groups. An SVM is a learning model that performs classification by identifying the class-separating hyperplane that maximizes the distance between training data points in each class (i.e., support vectors). Originally developed for binary classification tasks, the two-class SVM formulation can be extended to solve *k* > 2 classification problems by constructing *k* binary classifiers [23]. In this study, a one-versus-all (OVA) multiclass extension was employed. We considered three binary classifications among the control, SS, and LS groups: Control versus all other groups (C-SVM), SS group versus all other groups (SS-SVM), and LS group versus all other groups (LS-SVM).

Given an input dataset $x_{i}\;(i=1,\ldots ,n$; $x_{i}\in {\mathbb{R}}^{D}$), the decision function of the *r*th SVM classifier (*r* = 1, 2, 3) can be formulated as follows:

(1) $$f_{r}\left(x\right)=w_{r}^{T}\cdot x_{i}+b_{r}\;$$

where $w_{r}={\left(w_{r1},w_{r2},\ldots ,\;w_{rD}\right)}^{T}$ is the *r*th weight vector, and *b* is a bias. The intent of the *r*th SVM classifier is to minimize the following optimization problem:

(2) $$\mathrm{minimize}\;\frac{1}{2}{||w_{r}||}^{2}+C\sum \xi_{i}^{r}\;$$

(3) $$\mathrm{subject}\;\mathrm{to}\;y_{i}^{r}\left(w_{r}^{T}x_{i}+b_{r}\right)\geq 1-\xi_{i}^{r}\;$$

$$\xi_{i}^{r}\geq 0,\;i=1,\ldots ,n$$

where *C* is the penalty parameter. After each of the *k* binary OVA classifiers [$f_{1},\;f_{2},f_{3}$] was determined, the class of sample *x* (corresponding to the maximum value of the *k* binary classifier) was predicted as follows:

(4) $$y_{i}=argmax_{r=1,\;2,3}f_{i}\left(x\right)\;$$

We supplemented this SVM method with a recursive feature elimination (RFE) method to determine the optimal HRV feature set. A feature-ranking criterion is generated by feature scores, which are determined as the squared coefficient of feature *j* ($w_{j}^{2}$, *j* = 1, 2, … *D*) for a binary classification. Similar to the SVM method, the combined SVM-RFE method was originally limited to binary classification. In this study, feature-ranking scores were calculated on the three binary SVMs via OVA method. The final ranking score of the *j_th_* feature was determined as the maximum value of ranking scores among the three SVMs as follows:

(5) $$J_{j}=argmax_{r=1,\;2,\;3}\left({\left(w_{j}^{r}\right)}^{2}\right)\;$$

where $J_{j}$ is the cost for not selecting feature *j*, and $w_{j}^{r}$ is the weight-value that corresponds to the *j*th feature of an *r*th binary classifier. The feature with the smallest final ranking score was eliminated, and the classifier was retrained using the remaining feature set. This process was performed iteratively until a single optimal feature remained. During each iteration, the eliminated feature was reassigned to a lower ranking and the overall HRV feature-ranking criterion was recalculated.

#### 3.5.2. Optimal Feature Subset Determination

Determination of feature set were performed using different feature sets that were generated by iteratively including the highest ranked features based on the ranking criterion. The best feature subset was determined as that with the highest accuracy. The performance assessments were performed by using five-fold cross-validation of the data to ensure the integrity of this study’s results. The accuracy was determined as follows:

(6) ACC =(TN+TP)/(TP+TN+FN+FP) 

where *TP*, *FP*, *TN*, and *FN* represent the number of true positives, false positives, true negatives, and false negatives, respectively. In this study, various classification methods were considered to determine the optimal feature set including the SVM method, linear discriminant analysis (LDA), quadratic analysis (QDA), and the *k*-nearest neighbor (K-NN) algorithm. The SVM method included the use of linear, polynomial, and radial basis function (RBF) kernel functions.

### 3.6. Statistical Analysis

For statistical analysis, repeated measures analyses of variance (ANOVA) were conducted, followed by Tukey’s post-hoc tests, with group (control, SS, or LS) as the independent factor and time as the repeated measure. Subsequently, the effect of short- and long-term stress or control treatment on each parameter at different time points was further analyzed with a one-way ANOVA. The following *p* values were considered statistically significant: * *p* < 0.01 and ** *p* < 0.001. All data are expressed as mean ± standard error of the mean (SEM).

## 4. Results

### 4.1. Analysis of Effects of Short- and Long-Term Stress on HRV Features

**Figure 5:**
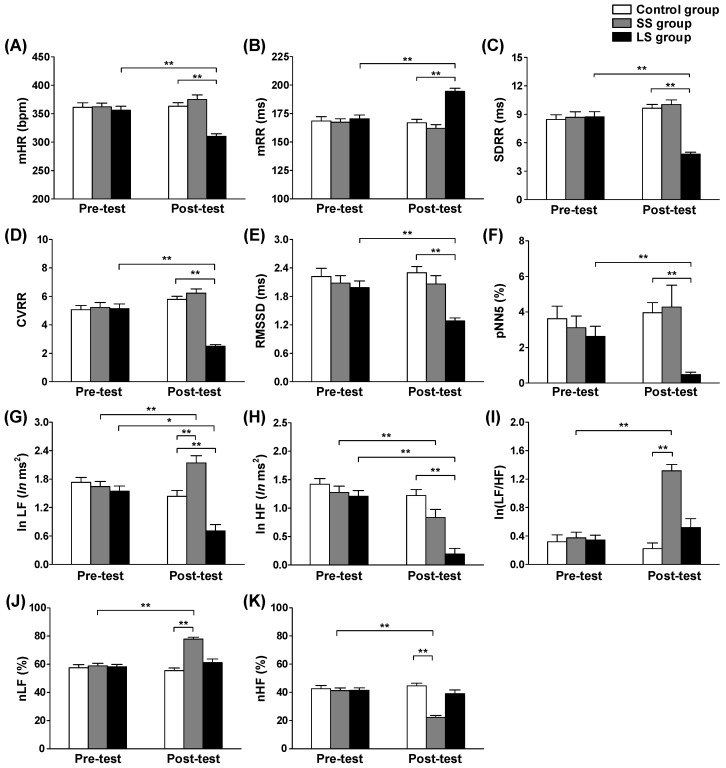
Effects of short- and long-term stress on (**A**–**F**) HRV time domain features and (**G**–**K**) frequency domain features. The LS group had significantly lower values across all HRV time domain features when compared with the control group and their pre-test. The SS group had significantly higher log-transformed low frequency power (*ln LF*), normalized low frequency (*nLF*), and log-transformed ratio of low frequency and high frequency powers (*ln (LF/HF)*) values and a significantly lower normalized high frequency (*nHF*) value when compared with the control group and their pre-test. (One-way analysis of variance followed by Tukey’s post-hoc test; * *p* < 0.01 and ** *p* < 0.001).

Before determining the optimal feature set, we investigated the effects of cumulated durations of stress exposure (short- and long-term stress) on various HRV features in both the time and frequency domains. Figure 5 compares these measured HRV feature values for the control, SS, and LS groups in pre- and post-tests.

Considering the HRV time domain features, when compared with pre-test, the LS groups showed increased *mRR* by 24.15 ms and decreased *mHR*, *SDRR*, *CVRR*, *RMSSD*, and *pNN5* by 45.82 bpm, 3.94 ms, 2.66, 0.7 ms, and 2.14%, respectively. Furthermore, the LS group had a significantly higher *mRR* value than that of the control group in the post-test (194.52 ± 2.72 vs. 166.82 ± 3.04 ms; *p* < 0.001). Conversely, the LS group had significantly lower *mHR* (310.29 ± 4.54 vs. 362.99 ± 6.33 bpm, *p* < 0.001), *SDRR* (4.82 ± 0.20 vs. 9.68 ± 0.40 ms, *p* < 0.001), *CVRR* (2.51 ± 0.11 vs. 5.81 ± 0.22, *p* < 0.001), *RMSSD* (1.28 ± 0.06 vs. 2.30 ± 0.13 ms, *p* < 0.001), and *pNN5* (0.48 ± 0.13 vs. 3.95 ± 0.57%, *p* < 0.001) values than that of control group in the post-test (Figure 5A–F).

Considering HRV frequency domain features, when compared with pre-test, the SS group showed decreased *ln HF* and *nHF* by 0.44 *ln* ms^2^ and 19.01%, respectively. In contrast, when compared with the pre-test values, the SS group had shown increased *ln* (*LF/HF*) and *nLF* by 0.94 and 19.01%, respectively. Furthermore, the SS group had a significantly higher *ln LF* (2.15 ± 0.15 vs. 1.44 ± 0.13 *ln* ms^2^, *p* < 0.001), *ln* (*LF/HF*) (1.32 ± 0.89 vs. 0.22 ± 0.78, *p* < 0.001), and *nLF* (77.81 ± 1.35 vs. 55.41 ± 1.90%, *p* < 0.001) value with lower *nHF* (22.19 ± 1.35 vs. 44.59 ± 1.90%, *p* < 0.001) values than that of the control group. When compared with the pre-test values, the LS group showed decreased *ln LF* and *ln HF* by 0.84 and 1.02 *ln* ms^2^, respectively (Figure 5G–K).

The results of the HRV analysis showed that the LS groups generally decreased in the time domain, whereas the SS groups had substantially significant differences in the frequency domain. Based on these results, both the HRV time and frequency domain features should be included to determine the optimal features for monitoring cumulative (short- and long-term) stress.

### 4.2. Determination of Optimal Feature Sets on Various Classifiers

**Figure 6:**
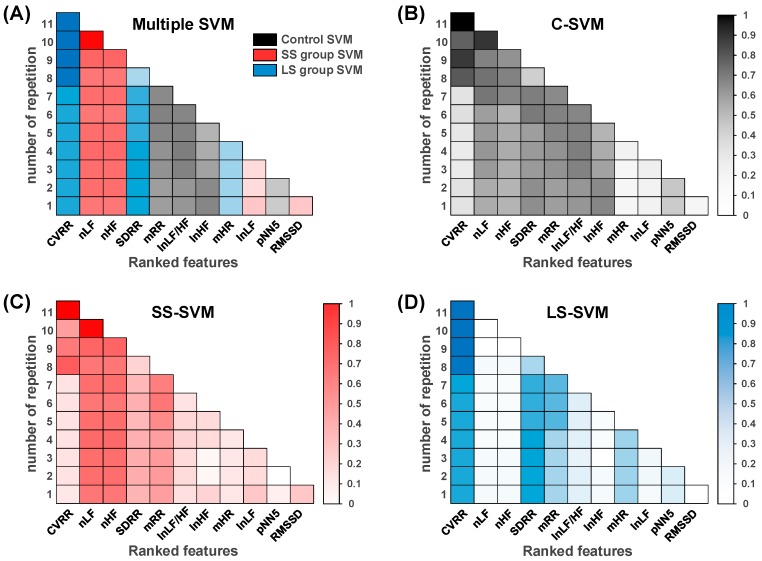
Colormap of weight value from the multiple SVM, Control versus all other groups (C-SVM), SS group versus all other groups (SS-SVM), and LS group versus all other groups (LS-SVM) for various HRV features. (**A**) The Multiple SVM included features determined by the C-SVM, SS-SVM, and LS-SVM. (**B**) Optimal features determined included the mean of R-R intervals (*mRR*), *ln (LF/HF*), and *ln HF* by the C-SVM; (**C**) the *nLF*, *nHF*, *ln LF*, and square root of the mean squared difference between adjacent R-R intervals (*RMSSD*) by the SS-SVM; and (**D**) the standard deviation of R-R intervals (*SDRR*), coefficient of variance of R-R intervals (*CVRR*), and mean of heart rates (*mHR*) by the LS-SVM. The results of the ranking process indicate the following descending order for the HRV features: *CVRR*, *nLF*, *nHF*, *SDRR*, *mRR*, *ln (LF/HF)*, *ln HF*, *mHR*, *ln LF,* proportion of N-N intervals that differ by more than 5 ms (*pNN5*), and *RMSSD*.

We obtained the feature ranking list and compared the optimal feature set based on various classifiers. First, the ranking criterion was generated for 11 HRV features for the control, SS, and LS groups using the SVM-RFE method. Figure 6 shows the colormap of the weight values in each iteration from the multiple SVM, C-SVM, SS-SVM, and LS-SVM for the 11 HRV features. The C-SVM was a binary classification that classifies the data into two classes: non-stress (Control) and stress states (SS and LS groups). The weight values of each iteration were normalized in the range 0–1 using w/‖w‖.

As shown in Figure 6A, the results of the ranking criterion were finally determined by multiple SVM, indicating the following descending order for the importance of HRV features considered in this study: *CVRR*, *nLF*, *nHF*, *SDRR*, *mRR*, *ln (LF/HF)*, *ln HF*, *mHR*, *ln LF*, *pNN5*, and *RMSSD*. The features with the highest and lowest order were *CVRR* and *RMSSD*, respectively. Each weight-value in the multiple SVM method was determined to be the maximum value among the C-SVM, SS-SVM, and LS-SVM classifications. Comparing the C-SVM, SS-SVM, and LS-SVM classifications in the first iteration, C-SVM had the highest *mRR* (0.39 vs. 0.38 vs. 0.30), *ln (LF/HF)* (0.41 vs. 0.14 vs. 0.11), *ln HF* (0.47 vs. 0.18 vs. 0.04), and *pNN5* (0.21 vs. 0.07 vs. 0.13) values, while SS-SVM had the highest *nLF* (0.33 vs. 0.54 vs. 0.04), *nHF* (0.30 vs. 0.54 vs. 0.04), *ln LF* (0.06 vs. 0.22 vs. 0.05), and *RMSSD* (0.05 vs. 0.23 vs. 0.01) values. Finally, LS-SVM had the highest *CVRR* (0.11 vs. 0.10 vs. 0.61), *SDRR* (0.44 vs. 0.31 vs. 0.63), and *mHR* (0.05 vs. 0.10 vs. 0.32) (Figure 6B–D)). In total, the multiple SVM method has generally selected the *mRR*, *ln (LF/HF)*, *ln HF*, and *pNN5* values from the C-SVM; the *nLF*, *nHF*, *ln LF,* and *RMSSD* values from the SS-SVM; and the *CVRR*, *SDRR*, and *mHR* values from the LS-SVM in whole iterations.

**Figure 7:**
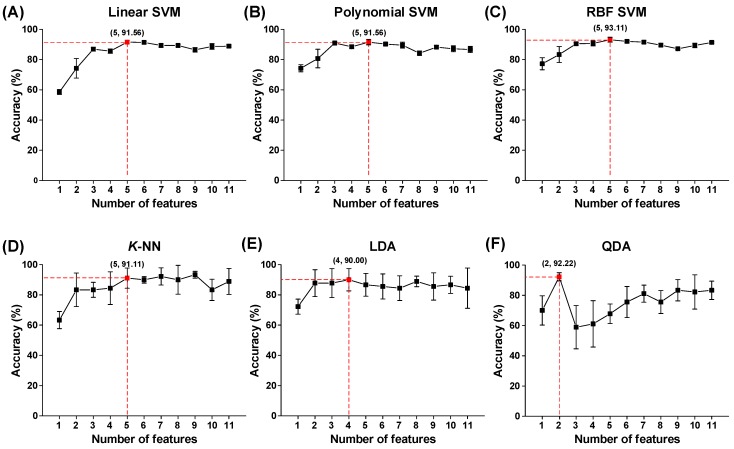
Comparison of optimal feature set on six different classifiers based on results of accuracies. Classifiers included (**A**) linear function SVM, (**B**) polynomial function SVM, (**C**) radial basis function (RBF) kernel function SVM, (**D**) *k*-nearest neighbor (*K*-NN), (**E**) linear discriminant analysis (LDA), and (**F**) the quadratic analysis (QDA) algorithm.

We determined the optimal feature subset of six classifiers (linear, polynomial, and RBF kernel SVMs; *K*-NN; LDA; and QDA) based on classification performances. Figure 7 shows the results of the classification accuracies according to the number of HRV features on the six classifiers. The linear, polynomial, and RBF kernel SVM classifiers achieved the highest performances on the top-five features (*CVRR*, *nLF*, *nHF*, *SDRR*, and *mRR*), obtaining 91.56 ± 1.13, 91.56 ± 1.80, and 93.11 ± 1.63% total accuracies, respectively. The *K*-NN achieved also the highest performance on the top-five ranked features, obtaining 91.11 ± 0.67% total accuracy. The LDA achieved the highest performance on the top-four ranked features, obtaining 90.00 ± 0.74% total accuracy. Finally, QDA achieved the highest performance on the top-two ranked features, obtaining 92.22 ± 2.72% total accuracy.

### 4.3. Comparison of Performances on Optimal Feature Sets

**Figure 8:**
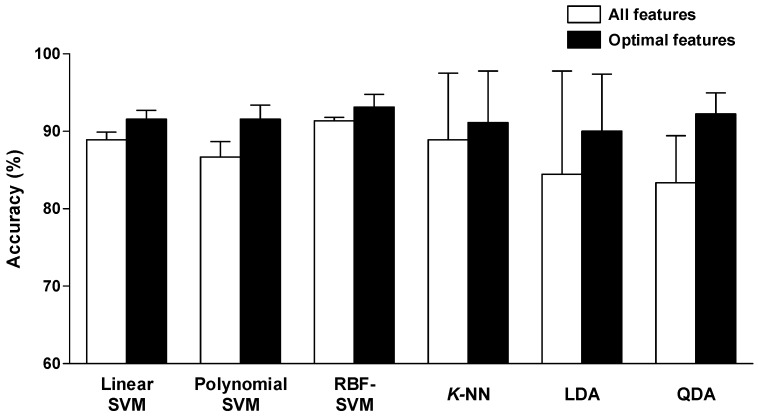
Comparison of six different classifiers based on overall accuracy for all HRV features and an optimal HRV feature set. The accuracy of each classifier increased when considering the optimal HRV feature set rather than all HRV features. The RBF kernel SVM classifier achieved the highest accuracy (93.11%) when considering the optimal HRV feature set.

We compared the performances of the optimal feature sets with those of all 11 features. Figure 8 shows the results of the accuracies for all 11 HRV features and for an optimal HRV feature set based on six classifiers. The accuracy for each of the classifiers improved when the optimal HRV feature set was considered. By considering the optimal HRV feature set rather than all HRV features, accuracy gains of 2.67, 4.89, 1.78, 2.22, 5.56, and 8.89% were observed for the linear, polynomial, and RBF kernel SVMs; *K*-NN, LDA, and QDA, respectively. The RBF kernel SVM classifier achieved the highest accuracy (93.11%) when considering the optimal HRV feature set. Based on these collective results, the RBF kernel SVM classifier proved most accurate when using an optimal HRV feature set comprising *CVRR*, *nLF*, *nHF*, *SDRR*, and *mRR*.

## 5. Discussion

In this study, we determined five optimal HRV features (the *CVRR*, *nLF, nHF*, *SDRR*, and *mRR*) for routine stress monitoring. Previous studies have similarly proposed optimal HRV features for stress monitoring [10,11,12,13,14,15,16]. However, most of those studies considered a singular exposure to a specific stressor. In daily life, light stress tends to accumulate over time. We therefore considered the effects of short- and long-term stress on various HRV features using a rodent model and determined an optimal HRV feature set for monitoring cumulative stress using SVM-RFE.

### 5.1. Results Interpretation

We first developed the cumulative stress model in animal, dividing the short-term (2 weeks) and long-term stress (4 weeks) in reference to the harmful effects of exposure to stress for more than 3 weeks. Based on our analysis of short- and long-term stress effects of different HRV time and frequency domain features, we found that HRV time domain features generally decreased under long-term stress, whereas HRV frequency domain features had substantially significant differences under short-term stress. The HRV time domain features reflect cardiac autonomic activation, whereas the HRV frequency domain features reflect ANS balance [5]. Thus, the results of this study suggest that short-term exposure to stress has no effect on ANS activity but causes a substantial ANS imbalance, shifting toward sympathetic activation. In contrast, long-term exposure to a mild stressor decreases ANS activity and causes a slight ANS imbalance. In other words, long-term stress led to reduced most HRV features, including features associated with both sympathetic and vagal tones, which indicated weakened overall ANS activities. However, in this study, the *mHR* decreased during long-term stress with a simultaneous increase in *mRR*. We can only assume that long-term stress dramatically decreased sympathetic activation compared with parasympathetic activation. Stubsjøen et al. [24] similarly found that stress exposure duration affected various HRV features. Specifically, they observed increased sympathetic activation and decreased vagal activity in sheep during days 9–17 but noted that these ANS tendencies adapted to chronic stress after day 17. The results of our study therefore contribute to the state of knowledge regarding the different effects of stress exposure durations on the ANS and suggest the need to consider both short- and long-term periods of stress in future research. Furthermore, the results of our study confirm the feasibility of using both time and frequency domain HRV features to detect or monitor daily stress by quantifying the activation of ANS.

When using the SVM-RFE method to determine an optimal HRV feature set, we found that the classification accuracy for each of the six different classifiers improved when an optimal HRV feature set was considered. For example, the RBF kernel SVM classifier proved to be the most accurate (93.11%) when using an optimal HRV feature set comprising *CVRR*, *SDRR*, and *mRR* as time domain features and *nLF* and *nHF* as frequency domain features. Among these features, *CVRR* and *SDRR* significantly decreased under long-term stress and were identified as optimal factors in LS-SVM. In general, *SDRR* reflects overall autonomic activity, and *CVRR*, which is the ratio between *SDRR* and *mRR*, indicates the total HRV without respiratory influence. A decrease in these features helps predict heart disease mortality [25]. Liu et al. [26] similarly identified *SDRR* and *CVRR* as optimal features for classifying chronic heart failure, which is known to be a stress-related disease.

Comparatively, both *nLF* and *nHF* were significantly different under short-term stress. These features represent the relative value of each power component in proportion to the total power [5]. In this study, the *ln LF* power was significantly higher for the SS group than for the control group with no significant difference in the *ln HF* power. Generally, *LF* (0.1 to 1.0 Hz range) is considered to be a marker of sympathetic activity [5] and has been found to increase in response to stress. Vanitha et al. [27] found significant differences in *nLF* across the four levels of stress. Because of this increased sympathetic output under short-term stress, *nLF* and *nHF* were identified as optimal factors in SS-SVM. Most studies used the *LF* or *LF/HF* of HRV for monitoring stress [28,29]. However, our results showed that *ln LF* and *LF/HF* were not selected as optimal features for monitoring stress. Similarly, previous studies had expressed doubt on the utility of *LF* and *LF/HF* features because *LF* and *LF/HF* were unsuitable for use as markers of sympathetic activity and autonomic balance, respectively [30], and may be affected by personal traits [7]. Our study suggested that *LF* and *LF/HF* cannot provide accurate information on cumulative stress.

The fifth ranked optimal feature in this study—*mRR*—helps in distinguishing between normal and stress states. Consistent with this study’s results, Boonnithi et al. [31] identified *mRR* as the best feature for distinguishing between rest and mental stress and achieved a classification accuracy of 79.9% by including this feature. In this study, although there are strong correlations between the *mRR* and *mHR* features, we observed different effects of *mRR* and *mHR* on feature selection because the relationship between the R-R interval and heart rate was non-linear owing to mathematical bias. In other words, the same changes in R-R interval resulted in high fluctuations of heart rate in a low average R-R interval than those in a high R-R interval [32]. For this reason, distributions within classes (control, SS, and LS groups) vary with respect to features. In this study, *mRR* had higher scores than *mHR* in the SVM-RFE method, especially for C-SVM. This finding showed that proper transformation is important for improving the feature’s importance in order to discriminate between classes.

### 5.2. Limitations and Future Works

In our study, we conducted an experiment to determine the optimal HRV features related with cumulative stress using an animal model. Prolonged exposure to stressful situations is not allowed in experiments on human beings due to strict ethical rules and personal variation [18]. Therefore, we used a stress animal model by applying the chronic mild stress procedure, which accurately mimics an environment with cumulated mild stress in daily life [33]. Over the last two decades, rodent models have been widely employed as useful tools for understanding the cardio-mechanisms in response to psychological changes [34]. Furthermore, since rat models have similar behavior to humans, various studies have used rats for investigating the effects of stress and depression [35]. However, rat models do have a different autonomic balance than humans because the ratio of unmyelinated to myelinated fibers of the vagus nerve in rats is higher than in humans [36,37]. The proposed HRV features obtained using rat models have some limitations in applications to stress monitoring systems for humans. However, we found the significance of HRV features according to cumulative stress in the animal model, and we appropriately designed the method for determining optimal HRV features for human stress monitoring systems using SVM-RFE.

In further studies, we will apply our method, which can determine the optimal features, to patients with stress-related disease and compare the results to the present animal model study. Furthermore, we will extend the experiment based on increased collection time of ECGs in consideration of individual’s physiological variation and recovery from the exposure to stress.

## 6. Conclusions

In this study, we investigated the effects of HRV features on cumulative stress, and found different changes in time and frequency domain features according to short- (two weeks) and long-term stress (four weeks). Based on this finding, the optimal features (*CVRR*, *nLF*, *nHF*, *SDRR*, and *mRR*) were determined with the highest performance of 93.11% on SVM-RFE. Our results demonstrate that the HRV is an effective method for monitoring the cumulative stress by assessing the activities and balance of the ANS, and the optimal features should be determined for an effective monitoring system. The HRV features determined in this study can help to facilitate development of in-facility and mobile healthcare system designs to support stress monitoring in daily life.
